# Dysregulation of miR-1-3p: An Early Event in Colitis-Associated Dysplasia

**DOI:** 10.3390/ijms232113024

**Published:** 2022-10-27

**Authors:** Mariana F. Fragoso, Geysson J. Fernandez, Lisa Vanderveer, Harry S. Cooper, Michael Slifker, Margie L. Clapper

**Affiliations:** 1 Cancer Prevention and Control Program, Fox Chase Cancer Center, Philadelphia, PA 19111, USA; 2 Group Biología y Control de Enfermedades Infecciosas, Universidad de Antioquia–UdeA, Medellín 050010, Colombia; 3 Department of Pathology, Fox Chase Cancer Center, Philadelphia, PA 19111, USA; 4 Biostatistics and Bioinformatics Facility, Fox Chase Cancer Center, Philadelphia, PA 19111, USA

**Keywords:** colitis, colitis-associated colon cancer, microRNAs, early detection

## Abstract

Detection of colorectal dysplasia during surveillance colonoscopy remains the best method of determining risk for colitis-associated colorectal cancer (CAC). miRNAs (miRs) show great promise as tissue-specific biomarkers of neoplasia. The goal of this study was to explore the miR expression profile of precancerous dysplastic lesions in the AOM/DSS mouse model and identify early molecular changes associated with CAC. Epithelial cells were laser-microdissected from the colonic mucosa (inflamed versus dysplastic) of mice with AOM/DSS-induced colitis. A miR signature that can distinguish inflamed non-neoplastic mucosa from dysplasia was identified. Bioinformatic analyses led to the discovery of associated miR gene targets and enriched pathways and supported the construction of a network interaction map. miR-1a-3p was one of the miRs with the highest number of predicted targets, including *Cdk6*. Interestingly, miR-1a-3p and *Cdk6* were down- and up-regulated in dysplastic lesions, respectively. Transfection of HCT116 and RKO cells with miR-1a-3p mimics induced apoptosis and cell cycle arrest in G1, suggesting its biological function. A slight reduction in the level of *CDK6* transcripts was also observed in cells transfected with miR-1. These data provide novel insight into the early molecular alterations that accompany the development of CAC and identify a miR signature that represents a promising biomarker for the early detection of colitis-associated dysplasia.

## 1. Introduction

Ulcerative colitis (UC), a type of inflammatory bowel disease (IBD), is characterized by episodes of active and quiescent inflammation that are limited to the colonic mucosa and extend from the rectum to the proximal colon [1]. The chronic inflammation experienced by patients with UC puts them at a higher risk of developing colon cancer than individuals who do not have colitis [2]. The cumulative risk of malignancy increases with the extent of colonic involvement, duration of disease, and severity of inflammation, with risk estimates reaching 18–27.5% after 30–45 years of UC [3,4]. Our current understanding of the early oncogenic events that accompany the transition from chronic inflammation to dysplasia, and ultimately cancer, remains limited.

Colitis-associated dysplasia, characterized by neoplastic epithelium confined to the basement membrane of the colon, is considered the best and most reliable biomarker of increased risk of malignancy [5,6,7]. However, early diagnosis of colitis-associated dysplasia remains challenging for several reasons: (1) lesions develop with a polypoid or flat morphology; as many as 50% of flat dysplasias in patients with pancolitis can be missed during surveillance colonoscopy [8]; (2) pathological identification of dysplasia on a background of inflamed mucosa is difficult and can lead to inter-observer variability in the diagnosis [9]; (3) clinical management of patients with low-grade dysplasia remains controversial, with a lack of consensus regarding the decision to undergo colectomy versus close surveillance [10,11,12]. Previous data from this group indicate that flat and polypoid colitis-associated dysplasia arise via distinct genetic mechanisms, and suggest that the establishment of a strategy for the comprehensive detection of dysplasia will require the identification of early biomarkers that are shared by both subtypes [13]. These results, when combined with the current guidelines of the American Association of Gastroenterology to initiate endoscopic screening 8 years after a diagnosis of UC [9], dictate the need to develop new strategies to detect colorectal dysplasia in this high-risk population earlier and with greater accuracy.

miRNAs (miRs), highly conserved noncoding RNAs (19–23 nucleotides), regulate gene expression post-transcriptionally by binding to the target sequence of mRNAs and either inhibiting translation or promoting protein degradation [2,14]. miRs are critical regulators of 30–60% of protein-coding genes in mammalian cells [15,16]. Because of their imperfect binding, a single miR can modulate the expression of hundreds of genes and impose control over many important cellular processes, including cell proliferation, differentiation, cell cycle progression, apoptosis, and embryonic development [5]. Characterization of the miR expression profile of several tumor types has led to the identification of specific miRs that are up- or down-regulated during tumorigenesis; indicative of their role as proto-oncogenes and tumor suppressor genes, respectively [6,7,17,18]. Data from genome-wide miR expression studies have confirmed that almost all cancer types exhibit a specific profile of miRs [19,20]. Therefore, miRs have been explored as a potential biomarker for cancer diagnosis and prognosis, and predicting response to treatment [21].

The mouse model of azoxymethane (AOM)/dextran sulfate sodium (DSS)-induced colitis represents a highly relevant system in which to investigate the molecular events associated with the formation of colonic dysplasias [22]. Similar to humans with long-standing colitis, immune competent mice experience periods of active and inactive disease and develop both flat and polypoid dysplasia/cancers. Many of the pathological features of murine acute and chronic colitis, including ulcers, crypt distortion, and infiltration of granulocytes, mimic those of human disease.

**Figure 1:**
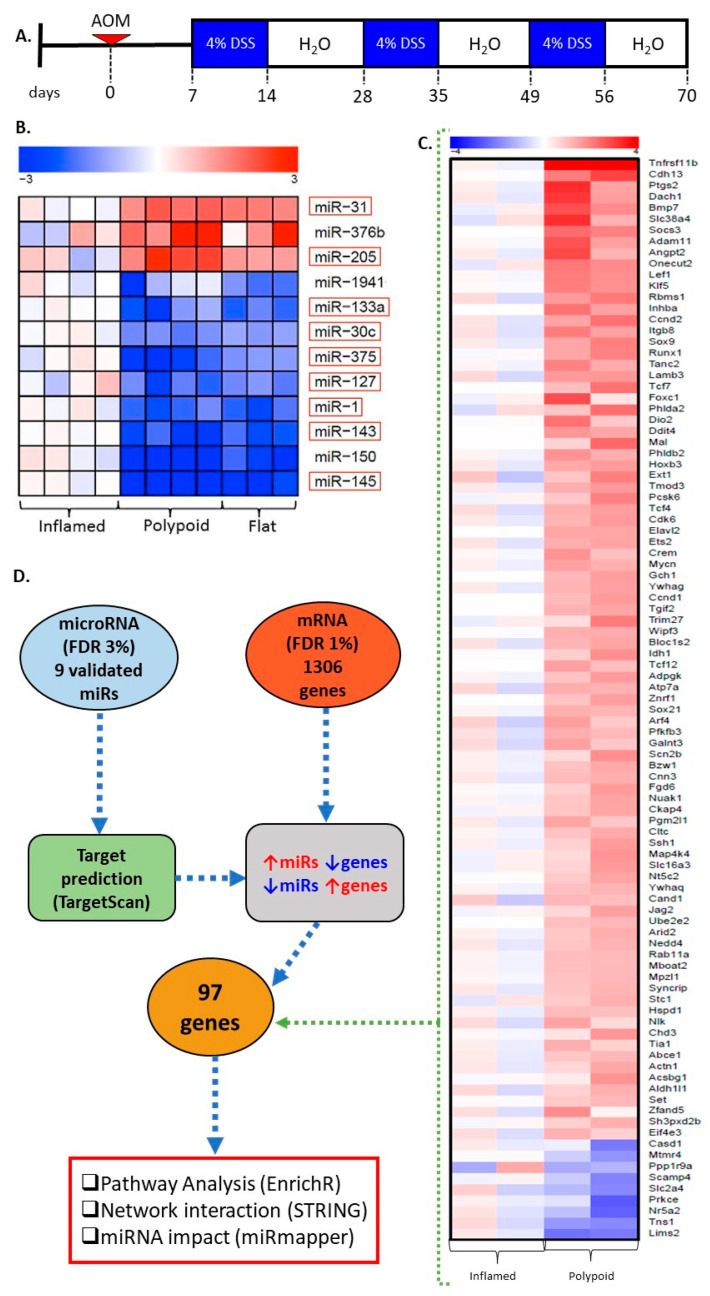
Identification of early events in the formation of colitis-associated colonic dysplasia. (**A**) Treatment regimen used to induce colitis-associated colorectal cancer in Swiss Webster mice. Animals were injected intraperitoneal once with azoxymethane (AOM, 7.4 mg/kg). Each treatment cycle consisted of 7 days of 4% DSS in the drinking water, followed by 14 days of untreated water. Animals were euthanized at the end of the last cycle (day 70). (**B**) miRs were profiled (FDR 3%) using the nCounter miRNA mouse assay (NanoString^®^). Red empty boxes—miRs validated by RT-qPCR; (**C**) Differentially expressed gene targets (n = 97) of the validated miRs that were subjected to in silico analyses (FDR 1%, *p* < 0.001); (**D**) Schema of the bioinformatics pipeline for pathway analysis, network interaction, and assessment of the biological impact of the miR signature. Data from in vivo studies (miRs and mRNA global expression) were subjected to bioinformatic analysis.

The goal of the present study was to explore the miR expression profile of precancerous dysplastic lesions in the AOM/DSS model of induced colitis (Figure 1A) to identify early molecular changes associated with the pathogenesis of colitis-associated colon cancer. Comparison of the global profile of transcripts and miRs in the non-neoplastic inflamed colon versus colitis-associated dysplasia led to the identification of a miR signature that has the potential to serve as an early biomarker of colitis-associated dysplasia. In addition, using in silico and in vitro approaches, we identified *Cdk6* as a target of miR-1a-3p (subsequently referred to as “miR-1”). To our knowledge, this study is the first to demonstrate the contribution of miR-1 dysregulation to colitis-associated carcinogenesis.

## 2. Results

### 2.1. miRNA Signature Differentiates Inflamed Non-Neoplastic Mucosa from Flat and Polypoid Dysplasia

Comparison of the miR expression profile of colonic epithelial cells microdissected from pathologically confirmed inflamed mucosa versus dysplasia led to the identification of 12 differentially expressed miRs (FDR 3%, *p* < 0.001) (Supplementary Data S1) (Figure 1B), 3 upregulated and 9 downregulated. Interestingly, no difference in the miR expression signature was observed between flat and polypoid dysplasias. An independent set of samples (inflamed = 8; flat = 6, polypoid = 8) was used to validate the differential expression of the 12 identified miRs by RT-qPCR. miRs were considered validated if they exhibited the same direction of change in expression (upregulated or downregulated) as the discovery set (n = 12). Nine of the 12 miRs were validated (2 upregulated and 7 downregulated) (red boxes in Figure 1B and Supplementary Table S1) and used for target prediction.

### 2.2. Genes Differentially Expressed in Colitis-Associated Colonic Dysplasias

Based on the predominance of polypoid lesions in the AOM/DSS model, the gene expression profile of inflamed mucosa versus polypoid dysplasia was compared. Numerous genes (N = 1094) were identified as differentially expressed (FDR 1%, *p* < 0.001) in inflamed versus dysplastic colonic epithelium (Supplementary Data S2) (Figure 1C). Fifty nine percent of these genes were up-regulated (771) and 41% (551) down-regulated in dysplasias versus inflamed mucosa. Pathways associated with the differentially expressed genes include Wnt signaling (MYC, CCND2), inflammatory response (PTGS2, CXCL10, IL1B) and apoptosis (IER3, JUN1, CD44). This gene expression signature was interrogated to inform the selection of specific miR targets for validation.

### 2.3. miR Targets Are Associated with the Progression of Colitis-Associated Carcinogenesis

The potential for the differentially expressed genes to serve as targets of the validated miR signature was explored. The use of TargetScan led to the identification of 3322 genes predicted to be targets of the nine validated miRs (Figure 1B, red boxes) (Supplementary Data S3). Only predicted target genes that were also differentially expressed, as determined by microarray, were retained for further analysis (N = 119 genes). The final list of genes subjected to pathway enrichment analysis (n = 97) was generated based on the contrasting up- or downregulation of the expression of the gene relative to the miR. For example, if the miR was down-regulated, only up-regulated target genes that were differentially expressed in the microarray data were analyzed further (Figure 1D). miRs without identified targets that met these criteria were omitted from further analysis (mmu-miR-127). The resulting 97 genes (Figure 1C,D) were used for the gene-term investigation in EnrichR, using enriched terms from KEGG Mouse 2019 and WikiPathways Mouse 2019 (Figure 1D). This same gene list was used to establish network interactions and identify the miRs with the largest potential impact (“hub miRs”).

**Figure 2:**
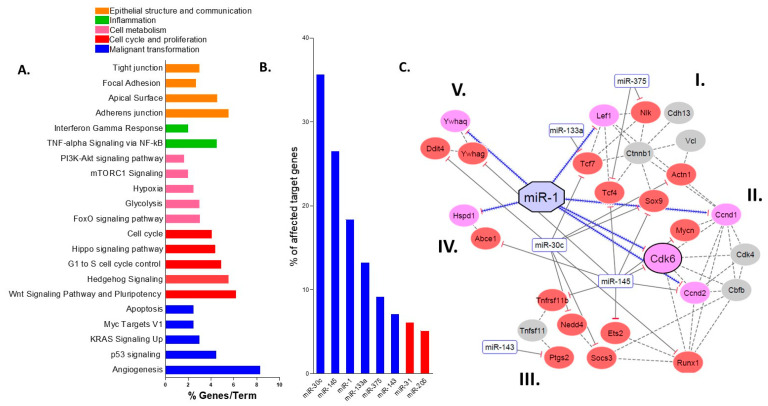
Bioinformatic analyses. (**A**) Enriched pathway analysis, illustrating the percentage of miR target genes per pathway, separated into 5 functional categories; (**B**) Ranking of top miRs in the identified signature based on the number of predicted target genes affected by their expression. Blue—downregulated miRs, Red—upregulated miRs; (**C**) Interaction network of genes and miRs (n = 48), predicted using the STRING database, depicting 5 protein–protein interaction clusters (I–V). Red circles—genes upregulated in the microarray profile; Gray circles—protein targets predicted from the STRING analyses; Pink circles—direct gene targets of miR-1; Blue rectangles, downregulated miRs from nCounter mouse miR (NanoString^®^) profiling; Blue contiguous arrows—targets of miR-1.

Gene ontology analyses revealed 21 terms associated with the pathogenesis and progression of UC and colitis-associated carcinogenesis (Figure 2A), including TNF-α signaling via NF-κB, G1 to S cell cycle control, Wnt signaling and apoptosis. These terms were divided into the following functional categories: (1) epithelial structure and communication; (2) inflammation; (3) cell metabolism; (4) cell cycle control and proliferation; and (5) malignant transformation. Thus, these results suggest the selected miR target genes have a significant impact on the biological functions that contribute to the progression of colitis-associated carcinogenesis.

### 2.4. miR-30c, miR-145a and miR-1 Are the Hub miRs of the Early Detection Signature

To better understand the ability of the validated miRs (n = 9) to coordinate the regulation of their target genes, an analysis of centrality was performed. Using this measure, miRs with the highest number of connections (hub miRs) with the selected targets (n = 97) were identified. miR-30c, miR-145a and miR-1 (“hub miRs”) were found to interact with the largest number of predicted gene targets (35%, 26% and 18%, respectively) (Figure 2B). Two factors were taken into consideration when selecting a predicted hub miR (miR-30c, miR-145 or miR-1) and its respective target gene for validation: (1) the number of clusters it regulated (Figure 2C), and (2) its known association with colitis-associated carcinogenesis.

### 2.5. Protein–Protein Interactions Categorize the Selected Targets into Five Functional Clusters

To further explore the enriched pathways, a protein–protein interaction (PPI) prediction was performed. Only genes associated with enriched pathways (Figure 2A) and predicted as targets of the miR signature (n = 47) were included in the PPI analysis (Figure 2C). Targets and miRs that did not meet these criteria (see in Material and Methods) were removed from the network.

Network interaction analyses led to the establishment of 5 different gene clusters (Figure 2C). In cluster I, miR-375 and miR-133a target *Tcf7* and *Tcf4*, downstream effectors of Wnt signaling and a pathway known to be dysregulated in colitis-associated cancer [23]. miR-375 also targets *Nlk*, a negative regulator of Wnt signaling [24], suggesting its protective role during colitis-associated carcinogenesis. In Cluster II, cell cycle control is primarily controlled by miR-1 (*Cdk6*, *Ccnd1* and *Ccnd2*) and miR-145 (*Myc*, *Cdk6* and *Ccnd2*). This cluster contains genes involved in the transition from the G1 to S phase of the cell cycle. Cluster III includes genes associated with inflammatory response, such as *Ets2* and *Ptgs2*. Interestingly, *Runx1* appears to connect Clusters II and III; previous reports indicate it can regulate epithelial cell proliferation [25] and inflammatory responses [26]. In addition, cluster III is targeted exclusively by miR-145, miR-143 and miR-30c, suggesting its direct role in inflammatory response. Cluster IV includes genes regulated by miR-1 and miR-145 and is associated with malignant transformation. For instance, the *Abce1* gene has been associated with promotion of cell proliferation, migration and invasion, as well as decreased apoptosis in several types of tumors, including lung cancer and oral squamous cell carcinoma [18,27]. Lastly, Cluster V is involved in cell metabolism and epithelial regeneration [28,29,30] via the mTOR pathway.

### 2.6. Biological Function of miR-1 In Vitro

miR-1 was selected for further evaluation based on its predicted ability to control several genes in each Cluster (I, II, IV and V). These clusters are considered clinically relevant because of their direct association with processes that are dysregulated early in the development of CAC: cell proliferation (I), cell cycle control (II), malignant transformation (IV) and the mTOR pathway (V). Unlike miR-145a, which is well documented in the literature as being involved in sporadic colorectal carcinogenesis [31], our current understanding of the role of miR-1 in CAC is very limited. Notably, hub miR-30c was not selected for analysis because it is predicted to regulate a smaller number of Clusters (I, III and IV).

### 2.7. miR-1 Induces Apoptosis in HCT116 and RKO Human Colon Carcinoma Cells

**Figure 3:**
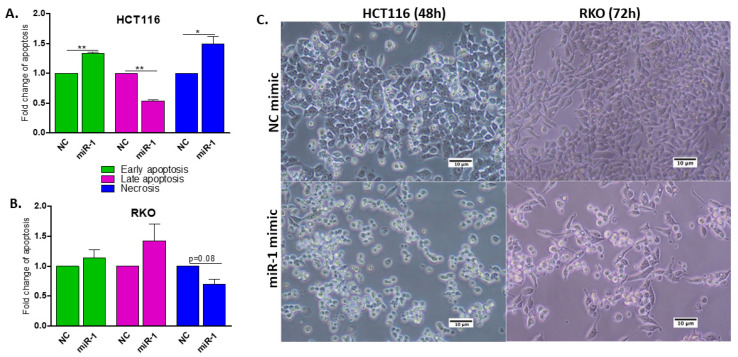
Effect of the miR-1 mimic on apoptosis in HCT116 and RKO cells (48 h and 72 h post-transfection, respectively). Fold-change in apoptotic cells was calculated by normalizing the percentage of apoptotic cells detected following transfection with the miR-1 mimic, to that of cells transfected with the negative control (cel-miR-67, NC) (mean ± SEM). Statistical comparisons were made using data from three experiments, expressed as log 10. (**A**,**B**) Analysis of cells undergoing early apoptosis (AnnexinV+), late apoptosis (AnnexinV+/7AA+), and necrosis (7AAD+) following transfection. (**C**) Representative images (10×) of RKO cells after transfection with cel-miR-67 (top) or miR-1 mimic (bottom), demonstrating the presence of rounded and floating cells. Comparisons significantly different by one-sample *t*-test (or paired *t*-test): * *p* ≤ 0.01, ** *p* ≤ 0.05.

Based on the top scoring enriched terms (Figure 2A), the effect of miR-1 on apoptosis and cell cycle progression was evaluated. Initially, HCT116 and RKO cells were reverse transfected with miR-1 mimics or cel-miR-67 (negative control) for 48 h. HCT116 and RKO cells treated with the miR-1 mimic had a higher proportion of early apoptotic cells, 30% and 20%, respectively, than cells treated with control miR mimic (*p* ≤ 0.01) (Figure 3A and Supplementary Figure S1A,B). When incubations were synchronized based on two doubling times (Doubling time: 23 h for HCT116 and 36 h for RKO), no effect on early apoptotic rates was detected in RKO cells (Figure 3B). However, a trend towards an increase in late apoptosis, followed by a decrease in the number of necrotic cells (*p* = 0.08), was observed. These data demonstrate that miR-1 induces apoptosis in both HCT116 and RKO cells after 48 h. This result provides evidence of the apoptotic function of miR-1 and suggests that the process of apoptosis is impaired in malignant cells during the progression of CAC, as indicated by the downregulation of miR-1 in dysplastic tissue.

### 2.8. miR-1 Induces Cell Cycle Arrest in G0/G1

**Figure 4:**
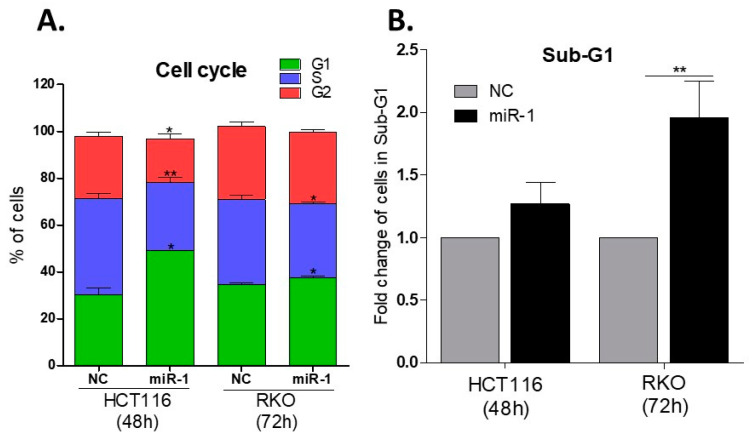
Impact of transfection with miR-1 on the cell cycle and sub-G1. (**A**) Effect of the miR-1 mimic vs. the negative control cel-miR-67 (NC) mimic on each phase of the cell cycle, demonstrating the miR-1 mimic increases the percentage of cells in G0/G1. Data are expressed as the mean percentage (± SEM) of cells in each phase and were obtained from 3 experiments. HCT116 and RKO cells were transfected for 48 h and 72 h, respectively. Unpaired *t*-test with Welch’s correction was used to compare the NC with miR-1 mimics for each phase of the cell cycle, in each cell line; (**B**) Fold-change of cells in Sub-G1 was calculated by normalizing the percentage of apoptotic cells detected following transfection with the miR-1 mimic, to that of cells transfected with the negative control (cel-miR-67, NC) (mean ± SEM). Comparisons were performed by one sample (or paired) *t*-test. * *p* ≤ 0.05; ** *p* ≤ 0.01.

FACS analyses revealed an increase in the number of cells in G0/G1 following treatment with miR-1 mimic (HCT116—49.2 ± 0.06% for miR-1 mimic vs. 30.3 ± 2.28% for NC); RKO (37.6 ± 0.59% for miR-1 mimic vs. 34.7 ± 0.58% for NC) (*p* ≤ 0.05), consistent with induction of cell cycle arrest by miR-1 (Figure 4A). Transfection of HCT116 cells with miR-1 mimic for 48 h decreased the number of cells progressing to S phase and G2/M significantly, as compared to cells transfected with the negative control (cel-miR-67) (*p* ≤ 0.01 and *p* < 0.05, respectively). Similarly, transfection of RKO cells for 72 h led to a reduction in the number of cells progressing to S phase (*p* ≤ 0.05), as compared to the negative control (*p* ≤ 0.05) (Figure 4A). Our data suggest that miR-1 induces cell cycle arrest in G0/G1 in both HCT116 and RKO cells, which contributes to the observed increase in cell death.

### 2.9. miR-1 Increases the Number of Cells in Sub-G1

To further assess the ability of miR-1 to induce cell death, the number of cells in Sub-G1 was evaluated by staining fixed cells with propidium iodide (PI), a known marker of late apoptosis and necrosis. Transfection with miR-1 mimic induced an increase in the number of RKO cells (*p* ≤ 0.01) in Sub-G1 (Figure 4B), as compared to cells transfected with the negative control (NC). Although a similar increase in cell number was observed in HCT116 cells, it did not achieve statistical significance (Figure 4B).

The effect of miR-1 on late-stage apoptosis was also confirmed by staining live cells with 7AAD, a marker that binds to exposed DNA in compromised membranes and discriminates late apoptotic and necrotic cells from live cells. Interestingly, the number of HCT116 cells stained with 7AAD (blue bars) was higher 48 h after transfection (Figure 3A) (*p* ≤ 0.05); an observation confirmed by the analysis of the Sub-G1 population (Figure 4B). A similar trend of an increased number of cells in Sub-G1 (Figure 4B) and late apoptosis (Figure 3B, pink bars) was seen in RKO cells overexpressing miR-1.

In summary, these data suggest that RKO cells begin to die as early as 48 h following miR-1 transfection, and progress to late-stage apoptosis and necrosis by 72 h. The presence of necrotic HCT116 cells at 48 h post-transfection suggests that miR-1 induces apoptosis in this cell line even earlier, prior to the second doubling (48 h).

### 2.10. miR-1 Targets the 3′UTR of Cdk6 in Colon Carcinoma Cells

**Figure 5:**
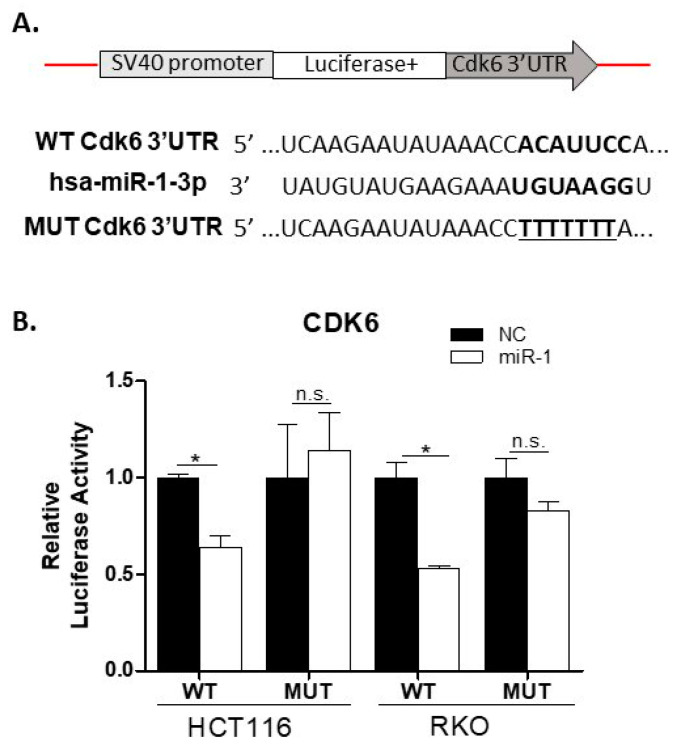
miR-1 is a direct target of *CDK6*. (**A**) Map of luciferase plasmid (top) and the predicted miR-1 binding site within the 3′UTR of CDK6 (bottom, bold). The sequence of the mutated 3′UTR of CDK6 is underlined; (**B**) Relative luciferase activity of HCT116 and RKO human colorectal carcinoma cells transfected with the miR-1 mimic or the negative control cel-miR-67 mimic (NC). The results are from three independent experiments, normalized to the NC, and expressed as the mean ± SEM. Statistical comparisons were performed using an unpaired two-tailed *t*-test with Welch’s correction. * *p* ≤ 0.05; “n.s.”—not significant (*p* > 0.05).

To further expand our knowledge of miR-1 regulation, we identified a specific miR-1 target for validation. Analysis of the Protein–Protein Interaction (PPI) led to the identification of 6 targets, 3 of which are involved in cell cycle regulation (*Cdk6*, *Ccnd1* and *Ccnd2*). *Cdk6* was selected for validation based on its involvement in inflammatory signaling [32] and reported association with ulcerative colitis [33] and colitis-associated dysplasia [34]. To validate the predicted interaction between miR-1 and *CDK6* (Figure 5A,B), dual-luciferase reporter assays were performed. As shown in Figure 5B, cotransfection of HCT116 or RKO cells with pGL3-CDK6-WT and miR-1 mimics led to a significant reduction in relative luciferase activity (30% and 47%, respectively, *p* ≤ 0.05). To confirm the specificity of the miR binding site on *CDK6* for miR-1, both cell lines were cotransfected with pGL3-Cdk6-Mut and a miR-1 mimic. No difference in relative luciferase activity was seen in either cell line when the mutant construct was used, suggesting the seed sequence was specific for the 3′UTR of WT *CDK6*. These data, when combined, confirm that *CDK6* is a target of miR-1.

**Figure 6:**
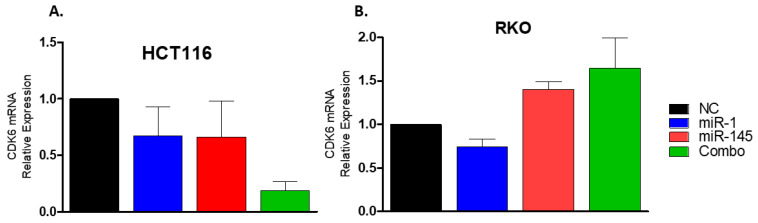
Levels of *CDK6* transcripts. The level of expression of *CDK6* mRNA was quantified by RT-qPCR in HCT116 and RKO cells 24 and 48 h, respectively, after transfection with miR (15 nM) (**A**,**B**). Cells were seeded and cultured in complete media for 24 h. The following day, cells were placed in media without FBS for two hours before transfection with miR-1, miR-145 or both miR-1 and miR-145 (combo) mimics. RNA was collected for *CDK6* expression analyses by RT-qPCR at each specific timepoint. Data from three experiments are expressed as mean expression (fold change) (±SEM), normalized to NC. Groups were compared using the Kruskal–Wallis test, followed by Dunn’s post hoc test. Significance = *p* < 0.05.

The ability of miR-1 to regulate expression of *CDK6* was further investigated by transfecting HCT116 and RKO cells with miR-1 mimics and evaluating expression of *CDK6* mRNA 24 h and 48 h later, respectively, by RT-qPCR (Figure 6A,B). Although statistical significance was not achieved, levels of *CDK6* expression were reduced (33%) in HCT116 cells transfected with either miR-1 or miR-145. Interestingly, cotransfection of HCT116 cells with both miR-1 and miR-145, another miR predicted to target *CDK6*, led to an 81% reduction in *CDK6* expression (Figure 6A). Although this result did not achieve significance (*p* = 0.09), it suggests that *CDK6* may also be targeted by miR-145 and that its reduction can only be observed when these miRs work in synergy, specifically in HCT116 cells (Figure 6A). Similarly, expression of *CDK6* was reduced (26%) in RKO cells transfected with miR-1, but no statistical difference was achieved (Figure 6B). In contrast to HCT116 cells, the combination led to an increase in *CDK6* expression; a result reinforcing that intrinsic differences between HCT116 and RKO cells can affect the way miRs regulate gene expression.

## 3. Discussion

To the best of our knowledge, the present study is the first to explore the role of miR-1 in the early stages of colitis-associated carcinogenesis. Use of in silico approaches to complement in vitro and in vivo model systems led to the: (1) establishment of a miR and mRNA global profile of colitis-associated dysplastic lesions in mice with AOM/DSS-induced colitis-associated colon cancer; (2) assessment of the biological function of miR-1 in vitro; (3) prediction of relevant miR target genes for validation; and (4) discovery of an interaction between miR-1 and the 3′UTR of *Cdk6*. These data indicate that the identified miR signature is able to distinguish colitis-associated dysplasia from the inflamed mucosa in mice with AOM/DSS-induced colitis. Associated molecular analyses revealed that miR-1 contributes to colitis-associated carcinogenesis by promoting cell cycle progression in transformed cells, as well as inhibiting apoptosis. These data suggest that *Cdk6*, alone or in combination with other genes, may represent a promising candidate for the early detection of colonic dysplasia in the context of UC.

Assessment of risk for CAC is currently restricted to the identification of dysplasia during surveillance colonoscopy, with no reliable molecular or genetic markers available for clinical use [12]. Patients with UC present with two morphological subtypes of colonic dysplasia: polypoid (visible with an elevated growth component), or flat (“invisible” and not elevated). Both arise from a background of chronic and dysregulated inflammation; an environment that promotes the initiation, development, and progression of CAC. Des-pite their current use, dysplastic lesions perform suboptimally as biomarkers of risk of CAC due to: (1). interobserver variability among endoscopists [9]; and (2) the challenge of identifying flat (invisible) lesions in numerous random biospies taken during surveillance colonoscopy [12]. Furthermore, clinical management of patients with low grade dysplasia remains controversial, with a lack of consensus regarding the decision to undergo colectomy [9]. Thus, identification of the molecular alterations that accompany the early progression of UC informs the design of interceptive interventions, as well as clinical treatment decisions for these high-risk patients.

Our understanding of the early biological and molecular events that drive the formation of colitis-associated dysplasia, including both flat and polypoid lesions, remains limited. To capture events that align with the window of opportunity for preventive intervention, the miR and mRNA expression profile of epithelial cells, microdissected from the inflamed and dysplastic colonic mucosa of mice with AOM/DSS-induced colitis, was compared. Surprisingly, the identified miR signature was differentially expressed in both flat and polypoid lesions, indicating its advantageous potential to serve as a pan bio-marker of colitis-associated dysplasia. Results from a previous study by this group demonstrate that flat and polypoid dysplasias arise via distinct molecular mechanisms, specifically loss of p53 and dysregulation of β-catenin, respectively [35]. Based on the presence of the identified signature in both types of dysplasias, dysregulation of these miRs must occur prior to the decision to develop as flat or polypoid, placing this event at the earliest stage of colitis-associated carcinogenesis. Thus, the resulting data enhance our understanding of the early molecular changes that occur within the inflamed colonic mucosa during progression to dysplasia.

While the contribution of miRs to colitis-associated colon carcinogenesis has been reported previously, these studies have focused primarily on late-stage tumor development [36,37,38]. For instance, miR-143 and miR-145 have been investigated extensively as tumor suppressor miRs in colorectal cancer [31] and were found downregulated in dysplastic lesions in our data. Relevant to the present study, transgenic villin-regulated pre-miR-143 and pre-miR-145 expressing-mice treated with AOM/DSS exhibited a reduction in tumor multiplicity, size and incidence [36,37]. In contrast and consistent with upregulation of miR-31 in the identified signature, overexpression of miR-31 has been observed in late-stage colon tumors in mice treated with AOM/DSS [38]. Furthermore, expression of miR-31 was elevated in high-grade dysplasias from patients with UC, as compared to the inflamed colonic mucosa [28]. Our data extend these previous findings by identifying a miR signature that is dysregulated early during colitis-associated carcinogenesis and remains altered throughout tumor development; an advantageous feature of a molecular target for preventive interception/early detection of CAC.

Attempts to establish an in vitro model system that recapitulates the inflammatory environment characteristic of UC have been met with limited success. This challenge was addressed in the present study by selecting two human colorectal carcinoma cell lines (RKO and HCT116) that exhibit oncogenic mutations similar to those found in CAC. According to Yaegar et al. (2016) [33], colitis-associated tumors often contain mutations and copy number variation in genes involved in RTK/KRAS signaling (57%), Wnt signaling, TGFβ and MYC (41%), cell cycle regulation (24%) and p53 signaling (83%). Likewise, both RKO and HCT116 cells possess mutations in *β-catenin* (Wnt signaling) [39], *ACVR2A* (TGFβ signaling), *PI3KCA* (PI3K pathway) and *ATM* (p53 signaling) [39]. In addition, RKO and HCT116 cells carry mutation of *BRAF* vs. *KRAS*, respectively (RTK/KRAS signaling) [39,40,41]. This similarity in mutational status enhances the applicability of the resulting data to the setting of colitis-associated carcinogenesis. Notably, RKO cells and HCT116 cells possess distinct polymorphisms within the proline-rich domain of *P53* (Arg/Pro 72 and Arg/Arg 72, respectively) [39]. The role of these polymorphisms in conferring predisposition to neoplastic transformation has been suggested but remains controversial [42]. With respect to functional activity, Arg72 appears to be a better suppressor of cellular transformation than Pro72, and superior in its ability to induce apoptosis [42]; an observation consistent with the extent to which the rate of apoptosis was increased in HCT116 and RKO cells following miR-1 transfection in the present study. Interestingly, *P53* Pro/Pro homozygosity has been associated with disease progression in patients with UC [43]. Therefore, even though these two cell lines exhibit many molecular features in common, the difference in *BRAF* vs. *KRAS* mutation as well as *p53* polymorphism may explain the contrasting effect of cotransfection with miR-1 and miR-145 on expression of *CDK6* (Figure 6).

The observed ability of increased miR-1 expression to enhance the rate of apoptosis and cell cycle arrest in G1 is consistent with its putative role as a tumor suppressor [44,45,46]. According to [47], ectopic over-expression of miR-1 induces cell cycle arrest and apoptosis in malignant mesothelioma cells by directly regulating genes associated with the apoptotic process. Interestingly, among the targets predicted in our in silico analysis, were 5 genes (*CASP9*, *MDM2*, *AKT1*, *IGF1* and *IFGIR)* directly associated with the intrinsic or mitochondrial pathway (Figure 2A and Supplementary Material). Although data from the present study provide insight into the biological function of miR-1, the manner in which this miR regulates the expression of genes associated with the apoptotic pathway remains to be elucidated. This study provides potential targets to be explored further.

It is important to note that cell death is an extremely dynamic process; not all cells progress through early versus late apoptosis/necrosis at the same rate, creating heterogeneity at the time of analysis [48]. Thus, the selected analytical method for quantifying apoptotic cells must consider the phase of the apoptotic process that is being targeted, as this can influence the interpretation of results derived from different methods [49]. When combined, our results demonstrate that miR-1 induces cell cycle arrest in G1, followed by cell death; a finding confirmed by the AnnexinV/7AAD apoptosis assay (Figure 3), analysis of the Sub-G1 population (Figure 4B and Supplementary Figure S1C), and morphological changes in both cell lines after transfection with miR-1 (Figure 3 and Supplementary Figure S1B).

Previous studies have shown that miR-1 is downregulated in sporadic colorectal tumors as compared to normal colonic mucosa [44], suggesting its contribution to late-stage colon carcinogenesis. Furthermore, downregulation of miR-1 within the colon of rats with acute TNBS colitis has been associated with worse disease symptoms and impaired epithelial barrier function [50]. Based on findings from the present study, decreased expression of miR-1 is expected to impair epithelial regeneration and delay the improvement of colitis symptoms. These data complement previous findings by extending the important tumor suppressive function of miR-1 to acute colitis and the early stages of colitis-associated carcinogenesis.

*CDK6* plays an important role in regulating the cell cycle, by forming complexes with D-type cyclins, such as cyclins D1, D2 and D3 [51]. The catalytic function of *CDK6*, its homologous enzyme *CDK4,* and the D-type cyclins facilitate phosphorylation of Rb, followed by release of the transcription factor E2F and progression from G1 to S phase of the cell cycle [32]. In addition, this protein has been reported to function as an inflammatory mediator [52] due to its direct association with p65 NFкB protein [53]. Therefore, the direct involvement of *CDK6* in cell cycle control and proliferation makes it a promising target for the prevention and/or treatment of inflammation-driven cancers and inflammatory diseases [52].

The observed upregulation of *Cdk6* in colonic dysplasias from mice with AOM/DSS-induced colitis, as compared to the surrounding inflamed mucosa, is consistent with clinical observations of altered *CKD6* expression in patients with UC [41,42]. Interestingly, genomic analyses of human CAC samples revealed that 24% of the UC-CAC cases had copy number alterations in a variety of cell cycle genes, including *CDK6* [33]. Thus, these observations: (1) identify *Cdk6* as an early molecular marker of colitis-associated progression, both in preclinical models and in humans with UC; and (2) confirm the clinical relevance of the AOM/DSS model of colitis-associated carcinogenesis.

A reduction in *CDK6* expression was observed in HCT116 (33%) and RKO (26%) cells following transfection with the miR-1 mimics; however, this change did not achieve statistical significance (Figure 6A,B). Several explanations for this result exist: (1) *CDK6* is only one of many genes involved in the highly regulated process of cell cycle control. For example, its association with D-type cyclins and binding to members of the INK4 family (p16^INK4a^, p15^INKb^, p18^INK4c^, p19^INK4D^) can positively or negatively impact its activity and expression, respectively [54]; (2) each miR physically interacts with hundreds of target genes to regulate gene expression in a global but integrated manner [55]. For instance, in our initial investigation, we predicted 665 genes to be targets of miR-1 (Supplementary Data S3). However, the efficiency of the miR function in regulating gene expression relies on its interaction with specific proteins to assemble the RNA-induced silencing complex (RISC) [56]. If too many targets are present in the cell, the efficacy of the miR-RISC machinery can be dissipated, reducing its activity [57]; and (3) to improve their efficiency, miRs often bind to the seed sequences in the 3′UTR of the same target gene in concert [56]. Indeed, a trend (*p* = 0.09) of this synergistic effect was observed in HCT116 cells transfected with both miR-1 and miR-145 (Figure 6A). These data provide novel insight into the ability of miRs to regulate gene expression through coordinated regulatory networks. Based on our emerging understanding of the dynamics and complexity of the mechanisms by which miRs regulate gene expression, an understanding of the comprehensive network, including the abundance of gene targets and other competing proteins, is required to accurately assess the functional significance of the differential expression of specific miRs.

## 4. Materials and Methods

### 4.1. Experimental Design

Female Swiss Webster mice (7 weeks of age) were purchased from Taconic Biosciences (USA) and housed in the Laboratory Animal Facility at Fox Chase Cancer Center (FCCC). Animals were maintained on Harlan Teklad 2018SX chow and received food and water ad libitum. At 8 weeks of age, mice were treated with AOM (7.4 mg/kg, single injection i.p.). One week later, all animals were administered 4% DSS in the drinking water for three cycles (one cycle = 7 days of DSS plus 14 days of untreated water) (Figure 1A). Following completion of the third cycle of DSS (at day 70), the animals were euthanized. Colons were excised and rinsed in PBS, with each half either fixed in 10% formalin for histopathological review and miR profiling, or embedded in OCT (global gene expression analyses). All animal procedures were performed in accordance with the guidelines of the Declaration of Helsinki and approved by the Fox Chase Cancer Center Institutional Animal Care and Use Committee (Protocol #06-16).

### 4.2. Laser Microdissection and RNA Extraction

For miR profiling, cells from the colonic mucosa were laser-microdissected from pathologically confirmed non-neoplastic inflamed mucosa (n = 4) or flat (n = 3) and polypoid (n = 4) dysplasias using a Leica LMD 6500 system (Leica Microsystems, Deerfield, MA, USA). RNA was extracted from the microdissected tissue using the RecoverAll™ Isolation kit (Life Technologies, Carlsbad, NM, USA). For global gene expression analyses, epithelial cells were laser-microdissected from non-neoplastic inflamed colonic mucosa (n = 2) and polypoid dysplasias (n = 2) using an AutoPix System (Molecular Devices, Sunnyvale, CA, USA). Total RNA was extracted using the PicoPure RNA Isolation Kit (Arcturus Biosciences, San Diego, CA, USA) and quantified using a Bioanalyzer 2100.

### 4.3. nCounter^®^ Mouse miRNA Expression Panel

miR expression profiling was performed using the nCounter^®^ mouse miRNA v1 expression panel (NanoString, Seatle, WA, USA). Total RNA was isolated from laser-microdissected inflamed mucosa (n = 4) or flat (n = 3) and polypoid (n = 4) dysplasias and analyzed for the expression of 577 miRs. Approximately 100 ng of each RNA sample was added to the miRNA-tag ligation reaction for annealing, followed by ligation and purification. Samples were diluted 1:10, and 5 µL of each (~10 ng total RNA) was added to 10 µL of Reporter Code Set, 10 µL Hybridization Buffer and 5 µL of Capture Probe Set for hybridizations. Quality control and normalization of raw gene counts was performed using NanoString nSolver^TM^ software v1 (NanoString, Seatle, WA, USA). Limma [58] was used to assess differential expression (log_2_-transformed normalized expression counts) between inflamed and dysplastic tissue samples. To adjust for multiple comparisons, false discovery rates (FDRs) were calculated from the resulting *p*-values via the Benjamini-Hochberg method [59]. Detailed information about this array can be obtained from the GEO database ncbi.nlm.nih.gov/geo, accessed on 30 November 2020, reference number GSE185504.

### 4.4. Global Gene Expression Microarray

The global gene expression profile of the inflamed colonic mucosa (n = 2) and polypoid dysplasias (n = 2) from animals with AOM/DSS-induced colitis was determined using Agilent 44K Whole Mouse Genome arrays (Agilent-012694). Array images were quantified with Agilent’s Feature Extraction v9.1 (FE) software, using Agilent Processed Signal (red and green) values as signal intensity measures. The R/Bioconductor package Limma [23] was used to fit linear models for all array probes. *p*-values from the empirical Bayes moderated *t*-tests were adjusted using the Benjamini-Hochberg procedure for controlling the FDR [24]. In the present study, only untreated colon tissue (inflamed mucosa versus polypoid dysplasia) was analyzed. Detailed information about this array can be obtained from the GEO database ncbi.nlm.nih.gov/geo, accessed on 30 November 2020, reference number GSE185599. The nonneoplastic mucosa is referred to as the “inflamed” mucosa throughout. 

### 4.5. Validation of miRNA Signature by RT-qPCR

Top scoring miRs (FDR 3%) were validated in an independent set of murine tissue samples (inflamed, n = 8; flat, n = 6; and polypoid n = 8) from AOM/DSS-treated mice. FFPE samples were laser-microdissected, as described previously, and RNA was extracted using the RecoverAll™ Isolation kit (Life Technologies, Waltham, MA, USA). For human colorectal cancer cells lines, RNA was extracted using TRIzol (Thermo Fisher, Waltham, MA, USA) according to the manufacturer. RT-qPCR was performed using the TaqMan miRNA Reverse Transcription Kit (Applied Biosystems, Waltham, MA, USA) for cDNA synthesis, and the TaqMan Universal PCR Master Mix No AmpErase UNG (Applied Biosystems, Waltham, MA, USA) for real-time PCR. The following TaqMan miRNA assays (Applied Biosystems, Waltham, MA, USA) were employed: mmu-miR-31, mmu-miR-376, mmu-miR-205, mmu-miR-1941, mmu-miR-133a, mmu-miR-30c, mmu-miR-375, mmu-miR-127, mmu-miR-1, mmu-miR-143, mmu-miR-150 and mmu-miR145 for mice and hsa-miR-1 for human cell lines. The expression level of each miR in inflamed versus dysplastic mucosa was measured using snoRNA202 and miR-23 (mice) and RNU44 (human) as reference controls. Fold-change was calculated, and miRs with a confirmed increase or decrease in expression were considered validated.

### 4.6. Target Prediction and Pathway Enrichment Analysis

Target prediction was performed on TargetScan (http://www.targetscan.org/vert_72/, accessed on 1 October 2020) using the validated, differentially expressed miRs (*p* < 0.001, n = 9) as input. The list of predicted target genes was compared with the list of differentially expressed genes from the microarray. A new list was generated that included target genes exhibiting changes in expression that were in contrast to those of their respective miR (e.g., downregulated miR and upregulated gene or vice versa). The resulting gene list was subjected to pathway enrichment analysis using EnrichR [60] (Accessed on 26 October 2020). Genes were assigned to pathways/functional categories, based exclusively on KEGG Mouse 2019, WikiPathways Mouse 2019 and MSigDB 2020. Pathways were initially filtered for general biologic processes associated with UC and CAC. A cut-off of 0.01 was applied to the adjusted z-score to filter enriched terms. A combined score of >15 was used to identify significantly over-represented functional categories (Supplementary Data S5).

### 4.7. Analysis of Centrality of the miR Signature and Construction of an Interaction Network

miRmapper was employed for centrality analysis, as is commonly done to define important players in disease or biological processes [61]. The resulting data were used to generate an interaction network using the STRING database (https://string-db.org/), accessed on 27 October 2020. This analysis provides a useful way to comprehensively group and organize protein-coding genes associated with complex biological processes [62]. Only genes associated with the enriched pathways were used for this prediction (n = 47). Interactions were calculated using “experiments” and “databases” as active interaction sources. The selection of interactions was set with a minimum score of 0.4. Cytoscape version 3.8.0 was downloaded from https://cytoscape.org/ (accessed on 30 November 2020) and was used to build the network.

### 4.8. Construction of 3′-UTR Reporter Plasmids and the Cloning Method

Oligonucleotides were designed based on the predicted ability of miR-1 to target the 3′ untranslated region (3′UTR) of *Cdk6* mRNA (Figure 3A). Sense and antisense sequences, corresponding to a ~50-bp fragment of *Cdk6* (*Cdk6*: position 8932-8985, NM_001145306), were synthesized based on information obtained from TargetScan v7.2 (http://www.targetscan.org/vert_72/), accessed on 30 November 2020. Oligonucleotides with a mutated target site served as negative controls. This modification was created by replacing the seed region of the miR site with a sequence of TTTTTTT [27]. The miR-1-specific targeting sequences (wild-type and mutants) were cloned into the pGL3 luciferase reporter plasmid at the XbaI site. Oligonucleotide annealing and pGL3-plasmid vector linearization were performed as described previously [63]. XL1-blue super-competent cells were used for transformation, and plasmid DNA was purified using the QIAprep Spin Miniprep Kit (QIAGEN). The correct orientation of the insert was confirmed by Sanger sequencing (Genewiz, South Plainfield, NJ, USA).

### 4.9. Cotransfection of Plasmid DNA, miRNA Mimics and pRL-TK Vector

HCT116 and RKO human colon carcinoma cells were cultured in McCoy’s 5a media (M4892, Sigma-Aldrich, St. Louis, MO, USA) and SMEM (Cat#11380-037, Gibco, ThermoFisher, Waltham, MA, USA), respectively, supplemented with 1% penicillin–streptomycin (MT#30-002-CI, Cellgro/Mediatech, Krackeler Scientifics, Albany, NY, USA), 2 mM L-glutamine (#MT25-005-CI, Cellgro/Mediatech, Krackeler Scientifics, Albany, NY, USA), and 10% fetal bovine serum (Cat#SH30071, Hyclone, South Logan, UT, USA) at 37 °C and 5% CO_2_. Cells were seeded in 24-well plates (0.5 × 10^5^ cells/well), and cotransfected with a luciferase plasmid containing the 3′-UTR of *Cdk6* (wild-type or mutant) and miR mimics (miR-1 and cel-miR-67) (miRvana™ miRNA mimics, Cat#4464070, Life Technologies, Waltham, MA, USA), along with the pRL-TK vector. Cel-miR-67 was used as the negative control for miR-1. Transfection was performed using Lipofectamine 2000 diluted in OPTI-MEM (Life Technologies, Cat#5198501). The working concentration was 2.5 ng/µL for PGL3 plasmid, 100 nM miRNA mimics, and 12.5 pg/µL pRL-TK. Cells were incubated with transfection medium for ≥2 h at 37 °C. After the incubation period, the transfection media was replaced with complete media, and the luciferase assay was performed 24 h later.

The effect of the miR-1 mimic on gene expression was determined using the Dual-luciferase Reporter Assay System (CAS: E1910, Promega, Madison, WI, USA), according to the manufacturer’s instructions. Firefly and renilla luciferase activity were recorded using a Berthold Technologies Microplate Reader (Centro XS3 LB 960, Dana Scientific Co., New York, NY, USA). The data were expressed as the ratio of firefly to renilla luciferase (normalized luciferase activity). Experiments were performed at least three times in triplicate.

### 4.10. Flow Cytometry Analyses for Apoptosis and Cell Cycle

HCT116 and RKO cells were seeded (0.5 × 10^6^ cells per well) and reverse transfected using Lipofectamine RNAiMax (Life Technologies, Waltham, MA, USA) with 15 nM mirVana™ miRNA mimics (miR-1 and cel-miR-67 as the negative control). After 48 h (HCT116) and 72 h (RKO), cells were harvested for apoptosis, cell proliferation and cell cycle analyses. The treatment duration for each cell line was equivalent to two doubling times (Doubling time: 23 h for HCT116 and 36 h for RKO) [64]. Dying/dead cells (AnnexinV+, AnnexinV+/7AAD+ and 7AAD+ only) were analyzed, following staining with APC AnnexinV and 7AAD (Cat #640930, Biolegend, San Diego, CA, USA), according to the manufacturer’s instructions. The cell cycle profile of each cell line was determined following fixation (70% ethanol overnight at 4 °C), using FXCyle PI/RNase (Cat #F10797, Invitrogen, Carlsbad, NM, USA) staining solution. Cells were sorted using BD FACSymphonyA5 and BD™ LSR II flow cytometers and data analyses were performed using Flowjo™ version 10.7.1 (BD, Ashland, OR, USA).

### 4.11. Cdk6 Expression in Human Colorectal Cancer Cells by RT-qPCR

HCT116 and RKO cells were seeded in complete media (0.5 × 10^6^ cells per well). After 24 h, media without FBS was added 2 h before transfection with cel-miR-67 (negative control), miR-1, miR-145 or the combination of miR-1 and miR-145 mimics. Transfections were done with Lipofectamine RNAiMax (Life Technologies, Waltham, MA, USA), and HCT116 and RKO cells were harvested 48 h and 72 h after plating, respectively. RNA was extracted using TRIzol (Invitrogen, Carlsbad, CA, USA), according to the manufacturer’s instructions. *CDK6* primers were synthesized by IDT (forward, 5′-TGGAGACCTTCGAGCACC-3′; reverse, 5′-CACTCCAGGCTCTGGAACTT-3′). RT was performed using the High-Capacity cDNA Reverse Transcriptase Kit (Applied Biosystems, Watham, MA, USA) for cDNA synthesis, and the iTaq Universal SYBR^®^ Green Super mix (BioRad, Hercules, CA, USA) for real-time PCR. The Ct method was used to calculate the fold-change in gene expression using HPRT as the reference gene [65]. Samples were normalized to the negative control for comparison.

### 4.12. Statistical Analyses

Statistical analyses for cell cycle, cell proliferation and luciferase assays were preformed using the unpaired *t*-test with Welch’s correction. For the apoptosis assay, one-sample *t*-test was performed. *Cdk6* expression was calculated using the Kruskal–Wallis test, followed by post hoc Dunn’s test. *p* < 0.05 was considered significant. Prism v5.01 was used for statistical analyses and preparing graphs.

## 5. Conclusions

In summary, a 12-miR signature has been identified that represents a promising biomarker for the early detection of colitis-associated polypoid and flat dysplasia within the inflamed colonic mucosa. Bioinformatic and molecular analyses led to the selection of miR-1 as a hub miR of the signature and confirmed its role in cell cycle progression and apoptosis. Downregulation of miR-1 and corresponding upregulation of *Cdk6* were identified in vivo as early events in the formation of colitis-associated dysplasia; alterations that may contribute to progression to CAC. Results from the present study provide unique insight into the pathological and molecular mechanisms associated with early stage colitis-associated carcinogenesis.

## Data Availability

The data presented in this study are openly available in GEO database at ncbi.nlm.nih.gov/geo (accessed on 30 November 2020), reference numbers GSE185504 and GSE185599. Additional data can also be found in the Supplementary Material.

## Supplementary Materials

The following supporting information can be downloaded at: https://www.mdpi.com/article/10.3390/ijms232113024/s1, Table S1: Validated miRs from the identified miR signature; Supplementary Data S1: miR expression data from inflamed tissue and flat and polypoid dysplasias; Supplementary Data S2: Microarray expression data from inflamed and polypoid dysplasia; Supplementary Data S3: miR target prediction results; Supplementary Data S4: Adjacency matrix between targets and miRs selected for network interaction; Supplementary Data S5: Pathway enrichment analysis. Figure S1: Effect of expression of the miR-1 mimic on apoptosis in RKO cells (48h post-transfection).
