# Supplementary material for: Dysregulation of miR-1-3p: An Early Event in Colitis-Associated Dysplasia
Source: Int J Mol Sci. 2022 Oct 27;23(21):13024. doi: 10.3390/ijms232113024 (PMC9657954; doi:10.3390/ijms232113024)
Supplement: Supplementary file 1 [file ijms-23-13024-s001.zip › Supplementary Figure S1- Fragoso et al.pdf]

Dysregulation of miR-1-3p: an early event in colitis-associated dysplasia

Fragoso et al.

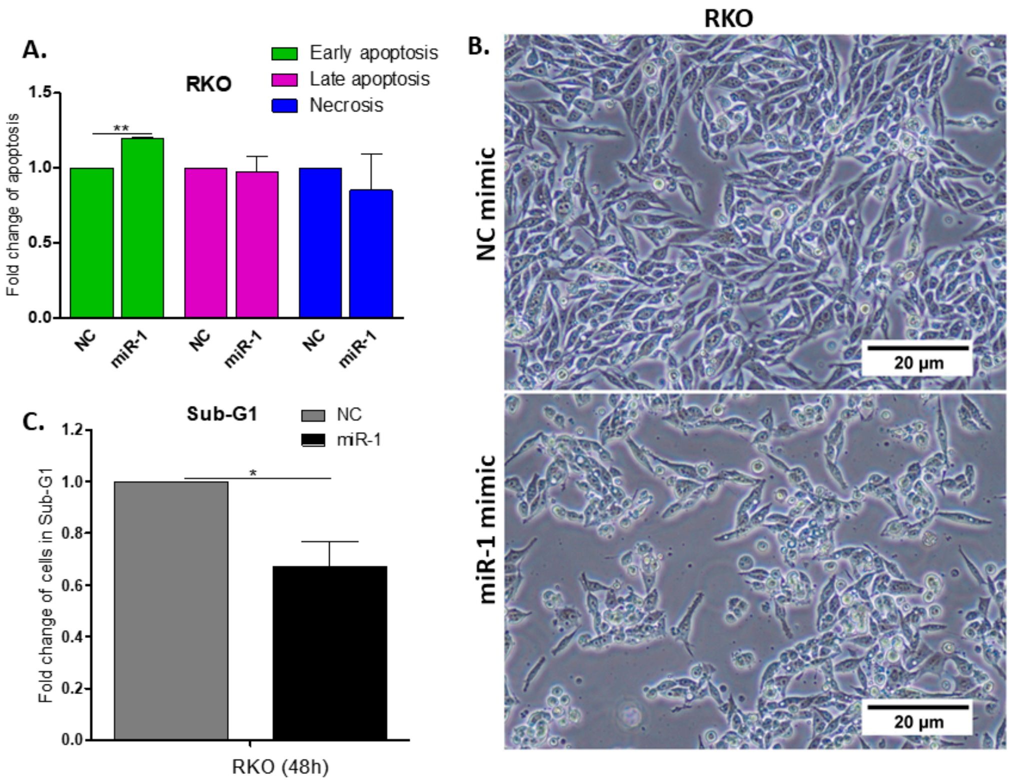

Supplementary Figure S1. Effect of expression of the miR-1 mimic on apoptosis in RKO cells (48h post-transfection). Fold change in apoptotic cells was calculated by normalizing the percentage of apoptotic cells detected following transfection with the miR-1 mimic, to that of cells transfected with the negative control (cel-miR-67, NC) (mean ± SEM). Statistical comparisons were made using data from three independent experiments, expressed as log 2. *A)* Analysis of cells undergoing early apoptosis (AnnexinV+), late apoptosis (AnnexinV+/7AA+), and necrosis (7AAD+) following transfection. *B)* Representative images (10x) of RKO cells after transfection with cel-miR-67 (top) or miR-1 mimic (bottom) for 48h, demonstrating the presence of round and floating cells. *C)* Fold change of RKO cells in sub-G1 48h after transfection with miR-1. Comparisons significantly different by one sample (or paired) t-test: * P≤0.05, ** P≤0.01.
