# Supplementary material for: Dysregulation of miR-1-3p: An Early Event in Colitis-Associated Dysplasia
Source: Int J Mol Sci. 2022 Oct 27;23(21):13024. doi: 10.3390/ijms232113024 (PMC9657954; doi:10.3390/ijms232113024)
Supplement: Supplementary file 1 [file ijms-23-13024-s001.zip › Supplementary Table S1-Fragoso et al.pdf]

Supplementary Table S1. Validated miRs from the identified miR signature.

**miRNAs**	**Fold Change (log2)**
miR-127	-0.251
miR-133	-0.174
miR-143	-0.728
miR-145	-0.817
miR-1a	-0.701
miR-205	1.339
miR-30c	-1.550
miR-31	3.457
miR-375	-2.328

miRs were validated by RT-qPCR using an independent set of murine samples (inflamed n=8; flat n=6; and polypoid n=8). Data are expressed as log2 fold-change. Positive and negative values indicate upregulation and downregulation of the indicated miR, respectively.
